# Anomalous Origin of the Left Common Carotid Artery from the Main Pulmonary Artery: A Rare Association in an Infant with CHARGE Syndrome

**DOI:** 10.1155/2016/2064937

**Published:** 2016-11-16

**Authors:** Onyekachukwu Osakwe, Blaise Jones, Russel Hirsch

**Affiliations:** ^1^The Heart Institute, Cincinnati Children's Hospital Medical Center, 3333 Burnet Avenue, Cincinnati, OH 45229, USA; ^2^Department of Radiology, Cincinnati Children's Hospital Medical Center, 3333 Burnet Avenue, Cincinnati, OH 45229, USA

## Abstract

*Case Report*. Isolated carotid artery originating from the pulmonary trunk is an exceedingly rare anomalous origin of head and neck vessels. We present this finding, along with a persistent embryonic trigeminal artery, in a male infant with multiple cardiac defects and other congenital anomalies associated with CHARGE syndrome. After extensive investigations, cardiac catheterization revealed the anomalous left common carotid artery arising from the cranial aspect of the main pulmonary artery. There was retrograde flow in this vessel, resulting from the lower pulmonary pressure, essentially stealing arterial supply from the left anterior cerebral circulation. The persistent left-sided trigeminal artery provided collateral flow from the posterior circulation to the left internal carotid artery territory, allowing for safe ligation of the anomalous origin of the left common carotid artery, thereby reversing the steal of arterial blood flow into the pulmonary circulation and resulting in a net improvement of cerebral perfusion.* Conclusion*. The possibility of this vascular anomaly should be considered in all infants with CHARGE syndrome. Surgical repair or ligation should be tailored to the specific patient circumstances, following a careful delineation of all sources of cerebral perfusion.

## 1. Introduction

Anomalous origin of head and neck vessels has been previously described, but specific origin of an isolated carotid artery from the pulmonary artery trunk is rare. We present this finding in an infant with CHARGE syndrome, who was also found to have persistence of the embryonic trigeminal artery, a finding that conferred some protection to the cerebral circulation.

## 2. Case Report

A male full term infant with multiple congenital anomalies was transferred shortly after birth to our hospital for further evaluation. At the time of transfer, he was known to have Ebstein's anomaly of the tricuspid valve, a moderately restrictive ventricular septal defect, small patent ductus arteriosus, and multiple other congenital anomalies, including bilateral choanal stenosis, cystic hygroma, microphallus, undescended testes, webbed neck, and dysmorphic facial features. Genetic evaluation confirmed a diagnosis of CHARGE syndrome.

His clinical exam on admission revealed a small, dysmorphic infant with a weight of 2.06 kg, heart rate of 160 beats per minute with equal peripheral pulses, blood pressure of 70/42 mmHg, respiratory rate of 50, and oxygen saturation of 100% on room air with a CPAP of 6. He was warm, well perfused, and comfortable with noninvasive respiratory support. He had a normal first heart sound with a physiologically split second heart sound, and a grade III/VI, high pitched holosystolic murmur best heard at the left upper sternal border. His abdomen was mildly distended, but his liver was not enlarged.

**Figure 1:**
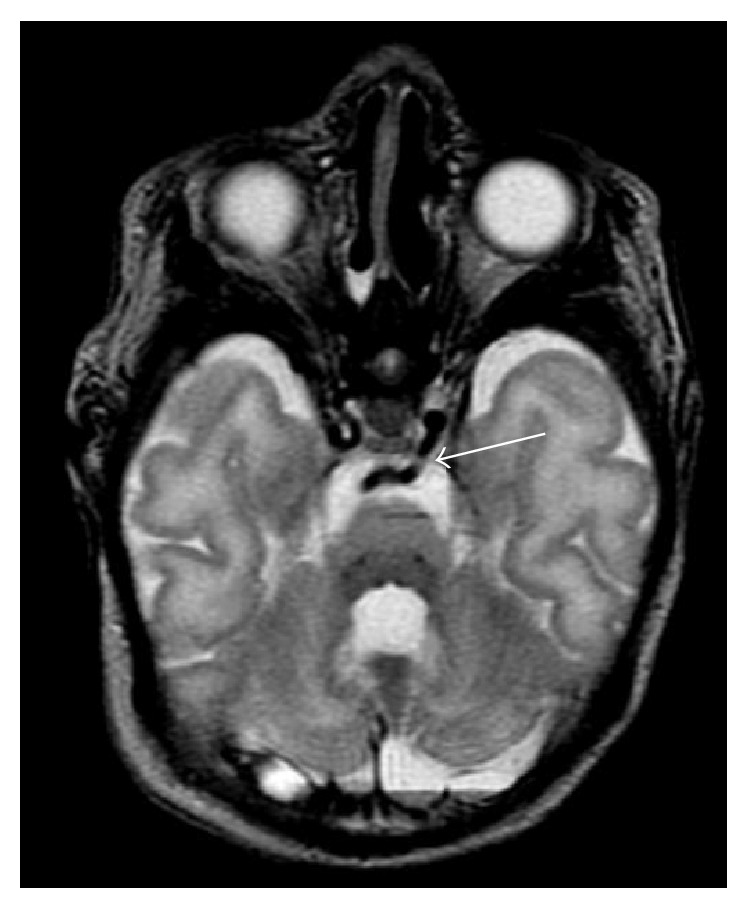
Axial T2-weighted MR image shows a persistent trigeminal artery (arrow) coursing from the cavernous left internal carotid artery posteriorly and medially to anastomose with the midbasilar artery.

His immediate evaluation included a chest X-ray that was consistent with a left-to-right shunt, with increased pulmonary vascular markings and mild cardiomegaly, but no focal infiltrates; an echocardiogram that revealed mild Ebstein's anomaly, mildly dilated right atrium, moderate perimembranous ventricular septal defect, bicuspid aortic valve, fenestrated atrial septum, a small PDA, a right sided aortic arch, and a poorly defined aberrant left subclavian artery; and baseline blood work with a hemoglobin of 10.3 g/dL. During the first 2 days after admission, he was able to be weaned from CPAP to blow-by supplemental oxygen, but his tachypnea persisted. Despite diuretic therapy, he would periodically require increased respiratory support on high flow nasal cannula for desaturation episodes. MR imaging of the brain and internal auditory canals was performed to evaluate for potential cochlear implantation in the future. It demonstrated a persistent trigeminal artery (TGA), an anomalous intracranial artery connecting the left internal carotid artery to the midbasilar artery (Figure 1). Genetic evaluation showed a heterozygous mutation in CHD7 (c.2514 T > A), which is pathogenic for CHARGE syndrome. There was no evidence of deletions of chromosome 22q11 on fluorescence in situ hybridization.

An invasive airway evaluation revealed tracheomalacia and bronchomalacia but no evidence of extrinsic compression. Over the course of eight weeks, with no indication of any improvement or change in his clinical status but with improved nutrition, it was decided to proceed with cardiac catheterization to better define the extent of his intracardiac shunt and help with decisions regarding reparative surgery. That procedure was performed from the femoral approach, with the patient under general anesthesia, and on room air. Saturation data indicated a pulmonary-to-systemic shunt ratio of 4.4 : 1 with a saturation step up from 43% in the superior vena cava to 77% in the left and right pulmonary arteries. Using measured oxygen consumption, the pulmonary and systemic blood flow were 6.74 and 1.53 L/min/m^2^, respectively. The left and right pulmonary artery saturations were identical. The right ventricular systolic pressure was 63% of the left ventricular systolic pressure, with the mean pulmonary artery pressure of 24 mmHg. The pulmonary vascular resistance was calculated as normal with a value of 2.74 Wu × m^2^.

**Figure 2:**
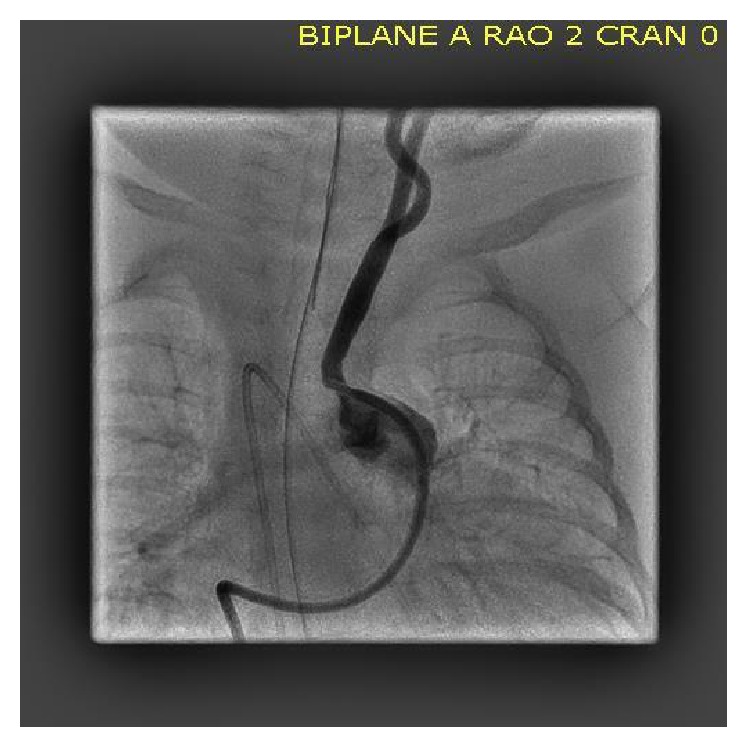
Camera in the AP plane. A contrast injection reveals anomalous origin of the left common carotid artery from the roof of the main pulmonary artery.

**Figure 3:**
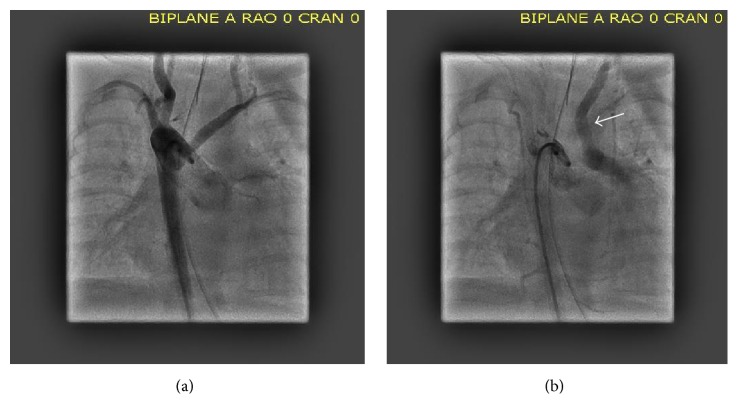
(a) Camera in the AP plane. An angiogram (early phase) is performed in the transverse aorta, revealing a right aortic arch with aberrant origin of the left subclavian artery and absence of the left common carotid artery. (b) Camera in the AP plane. An angiogram (late arterial phase) reveals retrograde opacification of the left common carotid artery (arrow) draining into the left pulmonary artery.

While attempting to advance the diagnostic wedge catheter (Cook Corporation, Bloomington In.) from the main pulmonary artery into one of the branches, it appeared that it was lodged in the roof of that vessel. A soft wire (0.018′′ HI-Torque Floppy, Abbott Vascular, Santa Clara, CA) was then passed through that catheter to attempt to gain access in one or other branches but instead passed easily in a cranial direction on the left side of the neck. The wedge catheter was then gently advanced over the wire, and a contrast hand injection was performed, revealing the anomalous left common carotid artery (LCCA) arising from the cranial aspect of the main pulmonary artery (Figure 2). Absence of an aortic origin of the carotid was confirmed with a supravalvar aortic angiogram (Figure 3(a)), demonstrating a right aortic arch with aberrant origin of the left subclavian artery, and the right common carotid artery arising as the first branch from the ascending aorta. Both vertebral arteries arose normally from the left and right subclavian arteries. In the late arterial phase of the injection, retrograde opacification of the LCCA was observed, draining into the branch pulmonary arteries (Figure 3(b)), reflecting preferential flow from the higher pressure cerebral arterial bed to the lower pressure pulmonary bed. The catheterization was completed without complication, and the infant was returned to the Neonatal Intensive Care Unit. One week later, the infant underwent uncomplicated surgical repair of the ventricular septal defect and atrial septal defect closure. Owing to the distance between the location of the anomalous carotid artery and the right aortic arch and concern for tension on the airway, the surgeon elected to ligate and release the anomalous vessel at the origin from the pulmonary artery. The infant has made an uneventful recovery from the procedure and made some progress weaning from ventilatory support but continued to require intensive care support.

## 3. Discussion

Anomalous origin of the carotid artery from the pulmonary artery trunk has been previously described but is exceedingly rare. Our literature review revealed a total of eight cases [1–8], of which two were in infants with conotruncal abnormalities [3, 5] and one in association with CHARGE syndrome [1]. One other infant was found to have other significant intracerebral arterial vascular anomalies in addition to the primary lesion [8]. The embryologic causation of aberrant arch vessels is multifactorial, with events that lead to either abnormal primitive arch regression or persistence of structures which should regress [1–7]. Persistence of structures may result in compression of adjacent structures (such as with vascular rings), while abnormal regression could impair blood flow and result in vessel hypoplasia or atresia. In the case of an aberrant carotid artery from the pulmonary trunk, a possible cause may be abnormal persistence of the left dorsal aorta between the third and sixth branchial arches, which becomes connected to the left pulmonary artery by the ductus arteriosus. The left third arch also regresses abnormally, which disconnects the left internal carotid artery from the other arch vessels [2, 4]. More recently, it has been proposed that this malformation may arise from poor septal formation of the aortic sac due to abnormal migration of cells from the neural crest at the cardiac level [7].

Trigeminal arteries are the most common of four described fetal arterial connections between the anterior and posterior cerebral circulations [9]. These anomalous connections normally involute at the 7 to 14 mm embryonic stage, with the only remaining arterial connections being the posterior communicating arteries [9]. The reported incidence of persistent TGA is 0.1–0.6% [9]. There is some decreased caliber of the basilar artery distal to the junction with the TGA in up to 75% of cases [10], but this feature was not evident in the presented case.

Anomalous origin of the LCCA has significant association with deletions of chromosome 22q11 (DiGeorge syndrome) [2, 5], but our patient was confirmed to have no evidence of deletion of chromosome 22q11. This case represents one in which an anomalous LCCA origin has been described with CHARGE syndrome. CHARGE syndrome has an incidence of 1 in 10,000 live births, and it presents variably with colobomas, heart defects, choanal atresia, growth and developmental retardation, and genital and ear abnormalities [11]. While often significant, the cardiac anomalies may not be the most important of the associated anomalies. However, when present, early repair may improve the physiology and allow the other comorbidities to be addressed on their merits. Often, as in our case, the impact or symptoms from the congenital heart lesion/s cannot be separated out from those caused by some of the other associated anomalies, and further evaluation is required. In this case, cardiac catheterization to determine the exact nature of the left-to-right shunt and calculate the pulmonary vascular resistance served as the vehicle to coincidentally diagnose the anomalous origin of the LCCA. Because of the lower pressure of the pulmonary circulation, there was retrograde flow in this vessel, effecting a “steal” of arterial supply from the left anterior circulation.

Surgical strategy to repair this defect is dependent on the extent of surgery required for reimplantation, the impact on adjacent structures after repair, and the physiologic impact of possible ligation if repair was deemed unlikely. The right aortic arch and aberrant left subclavian artery in this case resulted in the high likelihood of an extensive surgery to affect a repair, with significant tension and probable late stenosis. However, prior to proceeding with ligation during the planned congenital heart surgery, the integrity of the Circle of Willis and the presence of any other intracerebral vascular anomalies were deemed necessary, given those findings in one other case report. The left-sided TGA in this case provided robust collateral flow from the posterior circulation (basilar artery) to the left internal carotid artery territory, allowing for safe ligation of the anomalous origin of the LCCA. Not only did this ligation reduce the extent of required operative arterial manipulation but also it reversed the steal of arterial blood flow into the pulmonary circulation, resulting in a net improvement of cerebral perfusion.

## 4. Conclusion

Isolated origin of the carotid artery from the pulmonary trunk is a rare anomalous origin of head and neck vessels that may be more common in syndromic patients. Surgical repair or ligation should be tailored to the specific patient circumstances, although preceding evaluation in each case should include a careful delineation of all sources of cerebral perfusion. In addition to previously described cases in patients with conotruncal abnormalities, the possibility of this vascular anomaly should be considered in all infants with CHARGE syndrome.
